# Quantitative Representation of Autonomous Driving Scenario Difficulty Based on Adversarial Policy Search

**DOI:** 10.34133/research.0575

**Published:** 2025-01-17

**Authors:** Shuo Yang, Caojun Wang, Yuanjian Zhang, Yuming Yin, Yanjun Huang, Shengbo Eben Li, Hong Chen

**Affiliations:** ^1^ School of Automotive Studies, Tongji University, Shanghai 201804, China.; ^2^ Department of Aeronautical and Automotive Engineering, Loughborough University, Loughborough, Leicestershire LE11 3TU, UK.; ^3^ School of Mechanical Engineering, Zhejiang University of Technology, Hangzhou 310014, China.; ^4^ Frontiers Science Center for Intelligent Autonomous Systems, Shanghai 200120, China.; ^5^ School of Vehicle and Mobility, Tsinghua University, Beijing 100084, China.; ^6^ College of Electronics and Information Engineering, Tongji University, Shanghai 201804, China.

## Abstract

Autonomous vehicles with self-evolution capabilities are expected to improve their performance through learning algorithms, to automatically adapt to the external environment. However, due to the infinity, complexity, and variability of the actual traffic environment, it is necessary to develop quantitative representation indicators of scenario difficulty and generate targeted scenarios to ensure the evolution gradually, so as to quickly approach the performance limit of the algorithm. Therefore, this paper proposes a data-driven quantitative representation method of scenario difficulty. Specifically, the concept of environment agent is proposed, and a reinforcement learning method combined with mechanism knowledge is constructed for policy search to obtain an agent with an adversarial behavior. The model parameters of the environment agent at different stages in the training process are extracted to construct a policy group, and then agents with different adversarial intensities are obtained, which are used to realize data generation in different difficulty scenarios through the simulation environment. Finally, a data-driven scenario difficulty quantitative representation model is constructed, which is used to output the environment agent policy under different difficulties. Experimental results show the effectiveness of the proposed method. The result analysis shows that the proposed algorithm can generate reasonable and interpretable scenarios with high discrimination and can provide quantifiable difficulty representation without any expert logic rule design. Compared with the rule-based discrete scenario difficulty representation method, the proposed algorithm can achieve continuous difficulty representation. The video link is https://www.youtube.com/watch?v=GceGdqAm9Ys.

## Introduction

Autonomous driving technology has received more and more attention with the increasing level of automobile intelligence [1]. However, due to the challenges of algorithm level and technology maturity, there is still a distance to the realization of high-level autonomous driving. In recent years, self-evolutionary algorithms with experience storage and learning upgrade as the core idea have become a research hotspot [2–4]. Similar to human beings learning driving skills from limited scenarios to cope with infinite scenarios, autonomous vehicles with self-evolutionary algorithms are considered to have the potential to adapt to a huge number of scenarios in the real world and to realize a gradual improvement in performance [5–7].

Autonomous driving algorithms are required to undergo tens of millions of miles of data collection and testing before they can be deployed in real-world applications [8,9]. Self-evolving autonomous vehicles need to face enough scenarios and expose the algorithm’s problems, so this process is considered fundamental for the algorithm to achieve continuous self-improvement. However, the actual traffic environment is complex and it is difficult to be exhaustive, so it is necessary to obtain diverse, typical, reasonable, and interpretable environmental input information through scenario generation methods, so as to accelerate the efficiency of self-evolution and test evaluation [10,11]. There have been a lot of studies on related techniques, mainly including mechanism model methods and data-driven methods.

Scenario generation based on mechanistic models aims to generate and simulate scenarios that may be encountered by autonomous vehicles by utilizing experts’ understanding and professional experience of the changing rules within the scenario system, combined with logic rules and optimized solving. Rocklage et al. [12] proposed a method for automatically generating autonomous driving scenarios for regression testing. The method defines different types of traffic scenarios by creating static and mixed scenarios to guarantee a certain coverage of parameter combinations. de Gelder et al. proposed a scenario parameter generation method and a scenario representativeness metric. This method can determine enough parameters for scenario description and generate real parameter values by estimating the probability density function of these parameters. A Wasserstein-distance-based scenario representativeness metric is also proposed to quantify the realism of the generated scenario [13].

However, although mechanism model-based methods can make full use of existing knowledge to generate diverse and interpretable scenarios according to the test requirements, the artificial modeling method relies on a large number of expert experiences and various simplified conditions, which limits the practical application of these techniques. Compared with mechanistic modeling methods, data-driven methods have an obvious advantage because they can mine the potential laws from the data and fully consider the nonlinearities, uncertainties, and characteristic probability distributions in scenario generation [14,15].

Data-driven methods can be mainly divided into 2 categories, namely, natural scenario generation and adversarial scenario generation. Natural scenario generation aims to train a scenario generation model that is consistent with real driving conditions based on massive natural driving data through Bayesian networks [16], deep learning [17], and other methods. Diverse scenarios are generated by data-driven methods for training and testing of autonomous driving algorithms [18].

However, in natural scenarios, the probability of accidents for autonomous driving algorithms is very small, so it is necessary to construct targeted scenarios through adversarial scenario generation methods to approach the performance limit of algorithms quickly [19–21]. Zhang et al. [22] proposed a metamorphic testing framework for autonomous driving systems based on a generative adversarial network, which is combined with real-world weather scenarios to generate driving scenarios under various extreme weather conditions. Jia et al. [23] proposed a dynamic scenario generation method based on conditional generative adversarial imitation learning, in which scenario class labels are incorporated into the model to generate different types of traffic scenarios for inferring the weaknesses of the under-test autonomous vehicles. Rempe et al. [24] proposed STRIVE, a method that uses a learned traffic model to automatically generate realistic, challenging traffic scenarios that expose weaknesses in autonomous vehicle planners, ultimately enabling the improvement of these planners through targeted scenario generation. Hao et al. [25] proposed an adversarial safety-critical scenario generation method based on natural human driving priors, which uses human driving priors and reinforcement learning (RL) techniques to generate realistic safety-critical test scenarios covering both naturalness and adversarial.

It can be seen that the adversarial scenario generation method based on data-driven method can more effectively find the vulnerabilities of the algorithm and greatly accelerate the efficiency of algorithm training and testing. However, due to the black-box nature of data-driven methods, there are few studies that can generate interpretable and targeted quantifiable scenarios with different difficulty levels through explicit quantitative indicators of scene difficulty. However, the black-box nature of data-driven algorithms makes it difficult to accurately quantify and interpret the results of generated scenarios. This difficulty has led to few studies that clearly define quantitative criteria for scenario difficulty in order to generate interpretable and targeted quantifiable scenarios with different difficulty levels through explicit quantitative indicators of scenario difficulty.

In view of the above problems, this paper proposes a data-driven quantitative representation method of scenario difficulty for autonomous driving based on environment agent policy search. To our knowledge, this is the first proposed data-driven approach for quantifying scenario difficulty representation. The concept of environment agent is proposed, and an RL algorithm combined with mechanism knowledge is constructed to realize the policy search, so as to obtain the agent policy with an adversarial behavior. To obtain information on the quantitative dimension of scenario difficulty, the model parameters of environment agents at different stages of the training process are extracted into adversarial policy groups to obtain agent policies with different adversarial intensities. In the simulation environment, the data generation of the scenario with different difficulty levels is carried out and a scenario database is constructed. A data-driven scenario difficulty quantitative representation model is constructed, and the feature correlation of scenario input information is extracted through the attention network, and finally an environment agent policy that can output different difficulty scenarios is obtained. The proposed method can generate reasonable scenarios with high discrimination and can provide quantifiable difficulty representation without any expert logic rule design.

The contributions of this study are summarized as follows:

• This paper proposes the concept of environment agent, combines mechanism knowledge and the RL method to achieve efficient policy search, and obtains agent policies with adversarial behaviors.
• This paper proposes a data generation method for varying difficulty scenarios, which combines the policy groups constructed by model parameters at different stages in the training process to provide information on the quantitative dimension of scenario difficulty.
• This paper proposes a data-driven scenario difficulty quantitative representation model and proves that it can generate highly distinguishable scenarios with reasonable and quantifiable difficulty representations through result analysis.

This paper is organized as follows: The proposed framework is introduced in the “Proposed framework” section. The environment agent policy search is introduced in the “Environment agent policy search” section. The descriptions of data generation of varied difficulty scenarios are proposed in the “Data generation of varied difficulty scenarios” section. The details of quantitative representation of scenario difficulty are proposed in the “Experiment setting” section. In the “Simulation results” section, our method is verified and compared in simulation, and Conclusion concludes this paper.

## Results and Discussion

### Experiment setting

In this section, the proposed data-driven quantitative representation method of scenario difficulty for autonomous driving is validated in a simulation platform. The training scenario consists of randomly generated traffic flows with speeds ranging from 8 to 12 m/s within 180 m of the ego vehicle, as well as the surrounding vehicle with an adversarial behavior defined as the environmental agent. The training environment is constructed in the simulation software CARLA [26].

The soft actor algorithm consists of the value network and the policy network. The value network is a fully connected neural network with 3 layers, including 5 input neurons, 1 output neuron, and 256 hidden layer neurons. The policy network is a 4-layer fully connected neural network with 5 input neurons, 2 output neurons, and 256 hidden layer neurons. The scenario difficulty quantitative representation model φ consists of a transform decoder and a fully connected layer of 7 layers, where the number of neurons in the fully connected layer is 512.

The parameters of the algorithm are set as shown in Table 1. The parameter settings are subject to detailed selection and tuning. The simulation step size Δt=0.1s balances computational efficiency and simulation accuracy; the value network discount factor γ=0.99 ensures that future rewards are not overly discounted, promoting long-term decision-making; the learning rate of the adjust temperature $\lambda =3\times 10^{-4}$ and the learning rate of the target network $\tau_{1}=5\times 10^{-3}$ are fine-tuned through preliminary experiments to ensure stability and efficient convergence; the total number of training steps $n_{t}=100,000$ is determined based on the task complexity and observed convergence behavior; and the sampling interval k=10 balances the amount of training data used and computational efficiency.

**Table 1.** Hyperparameters for the simulation

Hyperparameters for the simulation	Symbol and value
Simulation step size	Δt=0.1s
Discount factor of the value network	γ=0.99
Learning rate of the adjust temperature	$\lambda =3\times 10^{-4}$
Learning rate of the target network	$\tau_{1}=5\times 10^{-3}$
Total training steps	$n_{t}=100,000$
Sampling interval	k=10
Same category data size	$n_{\mathrm{sc}}=10,000$
Episode number	$n_{\text{episode}}=1,000$
Batch size	$n_{b}=1,024$
Epoch number	$n_{\text{epoch}}=1,000$
Lane width	$L_{\text{lane}}=3.5\;m$

### Simulation results

The algorithm is deployed in the training environment. The computer is equipped with an Intel Core i7-10700 central processing unit and an NVIDIA GeForce GTX 1660 SUPER graphics processing unit. The ego vehicle’s policy is chosen as the learning-based autonomous driving algorithm with efficient self-evolutionary capabilities [3]. Specifically, the algorithm used is the advanced given algorithm accelerate-soft actor critic method, which combines behavioral cloning (BC) and soft actor critic (SAC) RL to optimize both efficiency and adaptability in complex traffic environments. This approach allows the ego vehicle to evolve autonomously by leveraging a mixture of suboptimal policy guidance and dynamic policy exploration, ensuring both accelerated convergence and enhanced adaptability to challenging driving scenarios.

#### Quantitative results

The effectiveness of the scenario difficulty generation method proposed in this paper is analyzed through quantitative results.

**Fig. 1.**
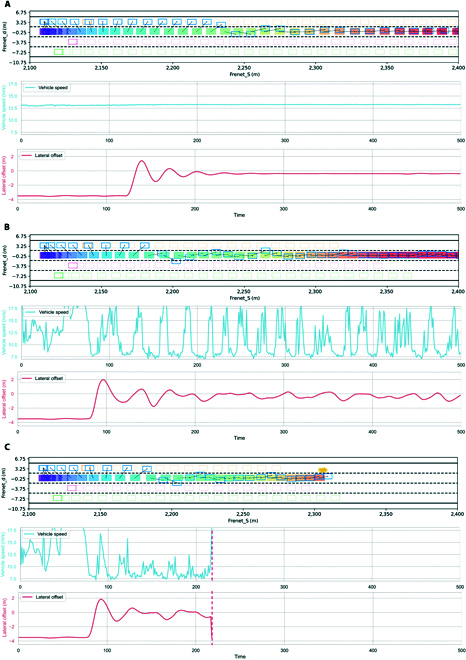
The trajectories, velocities and lateral displacements of the ego vehicle and the environment vehicles under various adversarial scenarios of different intensities. (A) Scenario difficulty factor $X_{\mathrm{scd}}=0.2$. (B) Scenario difficulty factor $X_{\mathrm{scd}}=0.5$. (C) Scenario difficulty factor $X_{\mathrm{scd}}=0.8$.

Figure 1 shows the trajectories, velocities, and lateral displacements of the ego vehicle and the environment vehicles under various adversarial scenarios of different intensities. Specifically, Fig. 1A to C present the results when the scenario difficulty factor $X_{\mathrm{scd}}$ is 0.2, 0.5, and 0.8, respectively. When the scenario difficulty factor is low, as in Fig. 1A, the speed change of the environment agent is smooth, resulting in little effect on the ego vehicle following behind. When the scenario difficulty factor $X_{\mathrm{scd}}=0.5$, as in Fig. 1B, the environment agent produces a more pronounced adversarial behavior. This is evident from the increased lateral offset, as it cuts in more aggressively while also generating sharp acceleration and deceleration to minimize the performance of the ego vehicle. In the most challenging scenario, the environment agent is able to actively resist the ego vehicle by jointly controlling the lateral offset and longitudinal speed until a collision occurs.

**Fig. 2.**
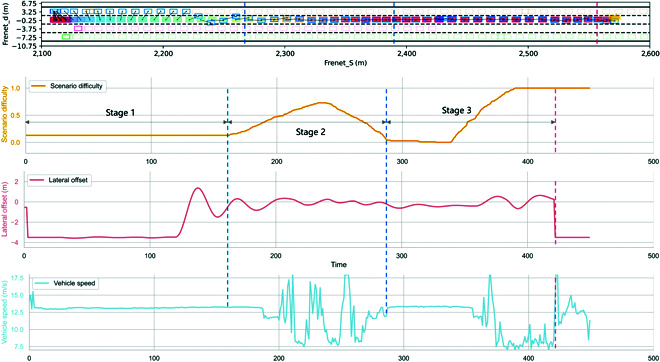
The trajectory, scenario difficulty, velocity, and lateral displacement of the ego vehicle and the environment vehicles under a continuously varying scenario difficulty factor.

Figure 2 shows the trajectory, scenario difficulty, velocity, and lateral displacement of the ego vehicle and the environment vehicles under a continuously varying scenario difficulty factor. The experiment is divided into 3 stages. In the first stage (stage 1), the scenario difficulty $X_{\mathrm{scd}}=0.13$. At this stage, the behavior of surrounding vehicles is relatively calm, having minimal impact on the ego vehicle, and the vehicle speed is relatively stable, indicating that the behavior generated under low-difficulty scenarios is reasonable and smooth. In the second stage (stage 2), the scenario difficulty gradually increases and then decreases. Surrounding vehicles periodically accelerate and decelerate to confront the ego vehicle, causing fluctuations in vehicle speed. In the third stage (stage 3), the scenario difficulty is raised to 1, reaching the maximum difficulty. The adversarial behavior of the surrounding vehicles is most intense at this stage, ultimately leading to a collision between the ego vehicle and the surrounding vehicles, verifying the reasonableness of the extreme behavior generated under high-difficulty confrontation scenarios.

#### Qualitative results

Through the qualitative results, the reasonability of the proposed method can be further analyzed.

**Fig. 3.**
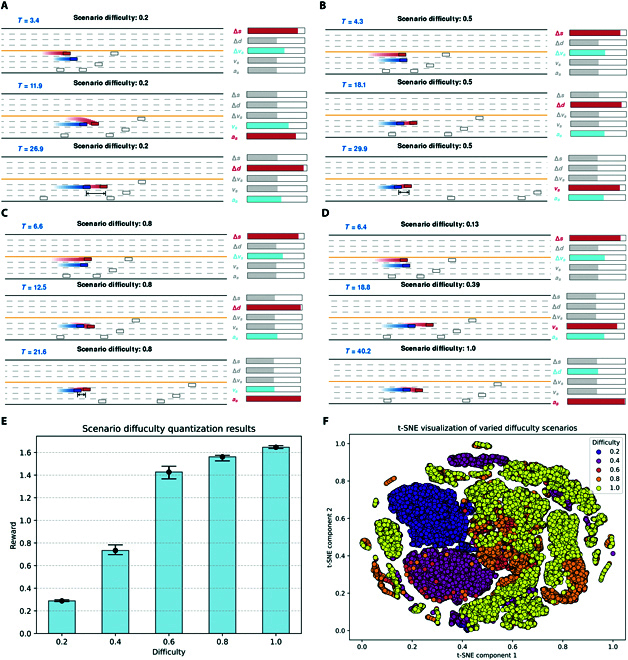
Visualization results of quantitative representation of scenario difficulty. (A) Scenario difficulty factor $X_{\mathrm{scd}}=0.2$, showing a moderate adversarial behavior. (B) Scenario difficulty factor $X_{\mathrm{scd}}=0.5$, showing an increased adversarial intensity closer to the ego vehicle’s limits. (C) Scenario difficulty factor $X_{\mathrm{scd}}=0.8$, showing an increased adversarial intensity closer to the ego vehicle’s limits. (D) Scenario difficulty factor $X_{\mathrm{scd}}$ that dynamically changes with manual input. (E) Scenario difficulty quantitation results. (F) t-distributed stochastic neighbor embedding (t-SNE) visualization of varied difficulty scenarios.

The visualization results of quantitative representation of scenario difficulty is shown in Fig. 3. Figure 3A to C illustrate the contribution ordering among input states for the fixed scene difficulty factor ($X_{\mathrm{scd}}=0.2,0.5,0.8$). It can be seen that as the scenario evolves, the change in the contribution ordering of state S consists of 3 phases:

1. Overtaking phase: the environment agent accelerates in order to reach the front of the ego vehicle to ensure that it can have an opportunity to influence the vehicle. At this point, state Δs has the highest contribution.
2. Cut-in phase: the environment agent in front of the ego vehicle generates adversarial behavior as much as possible through the control of the longitudinal movement. At this point, states $v_{s}$ and $a_{s}$ have the highest contribution.
3. Maintenance phase: the environment agent is in the process of confrontation with the ego vehicle. If there is any lateral offset deviation between the environment agent and the ego vehicle, the environment agent must block the ego vehicle and continue to produce adversarial behaviors. At this stage, state Δd has the highest contribution.

The above analysis process illustrates that the proposed data-driven quantitative representation method of scenario difficulty not only realizes the generation of variable difficulty scenarios but also has a certain level of interpretability compared with traditional black-box methods.

Figure 3D illustrates a scenario difficulty factor $X_{\mathrm{scd}}$ that dynamically changes with manual input. It can be seen that the proposed data-driven model can generate adversarial scenarios with continuous variable difficulty, and the correlation degree between states is also interpretable.

Figure 3E shows the quantization results of the scenario difficulty. The proposed scenario difficulty quantitative representation model is deployed to generate variable difficulty scenarios. The average reward for each episode is saved and used as a quantitative metric. It can be seen that the proposed method can generate diverse scenarios with quantifiable difficulty.

t-distributed stochastic neighbor embedding (t-SNE) is a machine learning algorithm for dimensionality reduction and visualization of high-dimensional data [27]. This method can be used to understand and present patterns and relationships in high-dimensional data. In this paper, the t-SNE method is applied to process the data in the varied difficulty scenario dataset. The t-SNE visualization of varied difficulty scenarios is shown in Fig. 3F. It can be seen that the scenario data of different difficulty levels have obvious aggregation. This shows that the proposed method can generate variable difficulty adversarial scenarios with discrimination.

## Conclusion

This paper proposes a data-driven quantitative representation method of scenario difficulty. The method constructed a variable-difficulty scenario generation model based on the transformer architecture through 2 key steps, namely, the policy search of the environment agent and the data generation of varied difficulty scenarios. The method was validated in a typical adversarial scenario. The experimental results demonstrated that the proposed algorithm can generate reasonable and highly distinguishable scenarios with quantifiable difficulty representations without any expert logic rule design. This applied to both fixed and dynamically changing scenario difficulty factor inputs. Furthermore, the analysis results indicated that the proposed method has a certain level of interpretability compared to traditional black-box methods. Future research will include large-scale natural driving datasets to generate more realistic adversarial scenarios. Additionally, the proposed method will be extended to cover more adversarial scenario generation tasks.

## Methods

### Proposed framework

**Fig. 4.**
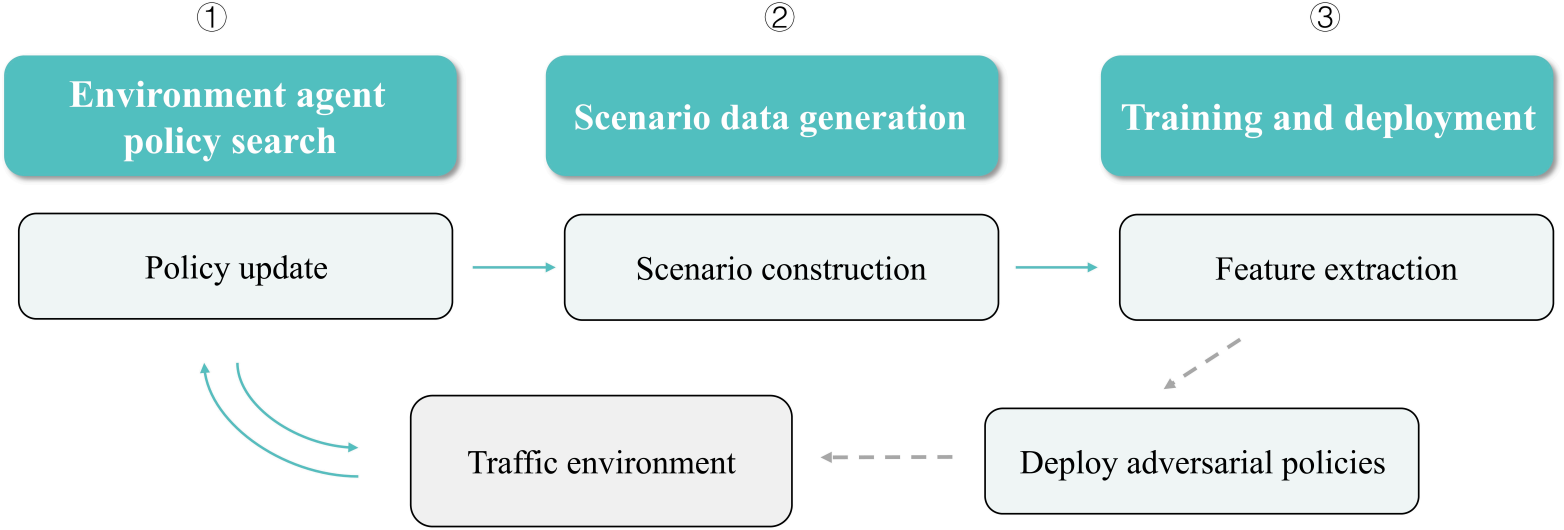
Overall architecture of the data-driven quantitative representation method of scenario difficulty.

As illustrated in Fig. 4, the proposed method comprises 3 main components: environment agent policy search, data generation for scenarios of varying difficulty, and training of a data-driven model to quantify scenario difficulty. First, environment agent policy search leverages RL to update policies and generate adversarial behaviors. Next, scenarios of varying difficulty are generated using models from different stages of training. Finally, a quantitative representation model is trained to extract feature associations from the scenario input data. The model inputs include the traffic environment information and a continuous scenario difficulty value, and the output is the action of the environment agent.

**Fig. 5.**
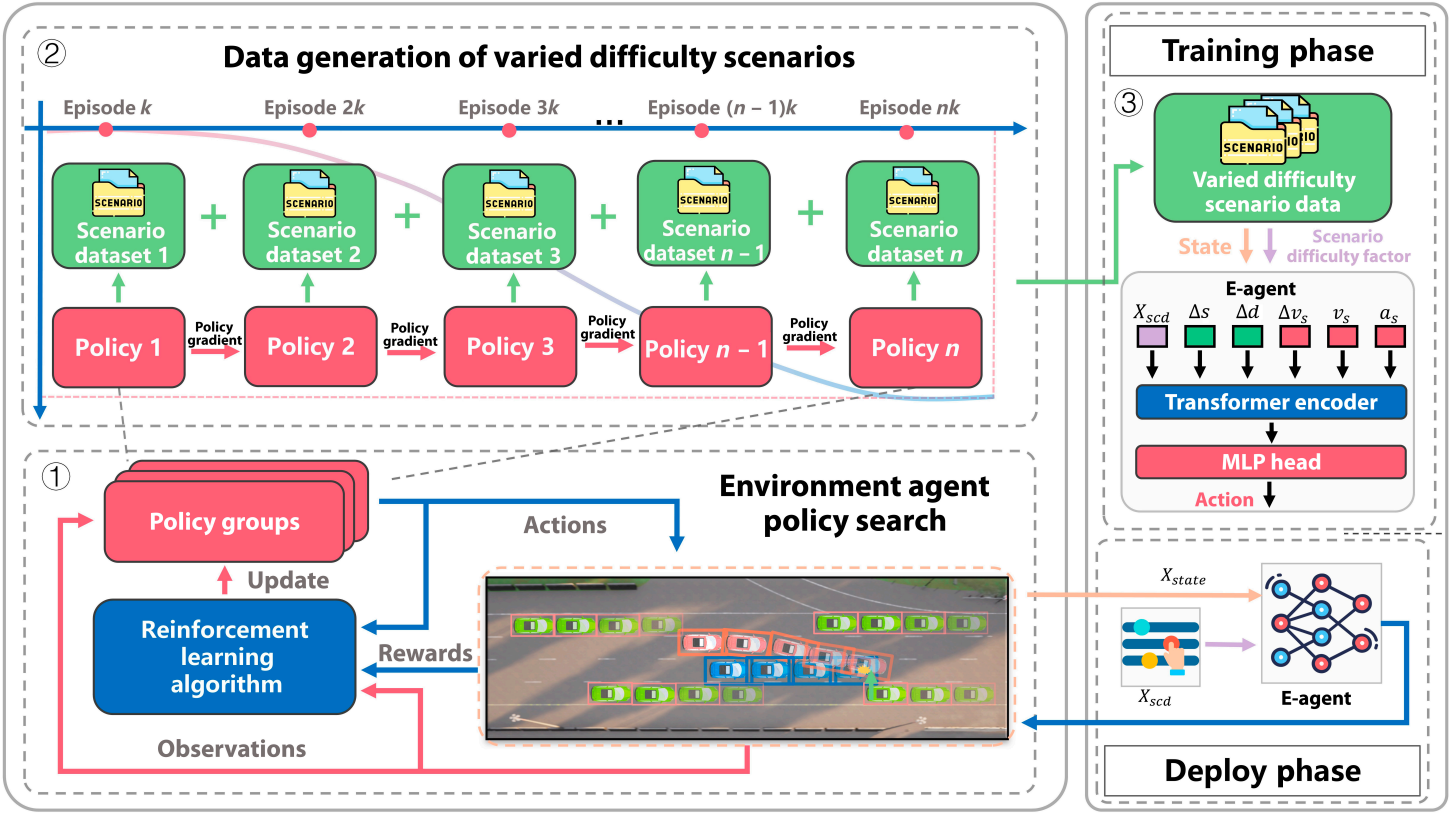
A detailed process diagram of the data-driven scenario difficulty quantitative representation method, including environment agent policy search, data generation of varied difficulty scenarios, and training and deployment phases of the scenario difficulty model. The process starts with policy search, generates scenario datasets of different difficulty levels, and then trains a quantitative representation model to extract scenario features and deploys the environment agent to create scenarios with continuous difficulty levels. MLP, multilayer perceptron.

This method employs a deep neural network as the environment agent and addresses the problem of adversarial policy search through an RL algorithm. As shown in Fig. 5, the model parameters trained at various stages are updated, saved, and output into the constructed policy group. The process of data generation varying difficulty scenarios involves 2 parts: deploying adversarial policies from the policy group and constructing the scenario database. Multiple policies from the policy group are successively deployed to the simulation environment for data generation of varying difficulty scenarios and appended to the scenario database. The proposed method mainly includes 2 phases: the training phase and deployment phase. In the training phase, the attention mechanism model is used to extract the feature association between the scenario difficulty factor and the state input. This allows for the creation of an environment agent policy capable of outputting different difficulty scenarios. Furthermore, in the deployment phase, the environment agent is deployed to generate scenarios with continuous difficulty levels.

### Environment agent policy search

#### Problem definition

Adversarial scenario generation aims to construct various complex, dangerous, or extreme traffic scenarios in order to expose the weaknesses of autonomous driving algorithms when facing different environmental inputs. Based on this, improving the performance of the algorithms based on the evaluation results can further improve the reliability and safety of the autonomous driving system.

In this paper, the concept of environment agent is presented for generating adversarial scenarios. The optimization objective of the environment agent policy is to maximize the influence of the scenario information input on the performance of the ego vehicle through the agent policy search. The optimization problem can be formulated as follows:

$$\theta^{*}=\underset{\theta }{\text{arg max}}\left[F\left(\pi_{A},\pi_{E}\left(\theta \right),S_{c}\right)\right],$$ (1)

where $F\left(\cdot \right)$ is the performance evaluation metric, $\pi_{A}$ is the ego vehicle policy, $\pi_{E}$ is the environment agent policy with parameter θ, and $S_{c}$ is the scenario dynamics. $S_{c}$ is used to characterize the state transitions of all agents.

The RL algorithm is dedicated to searching for optimal policies in order to maximize future returns. Therefore, this paper constructs an RL problem to realize the policy search for the above optimization problem. Considering that the adversarial behaviors of the environment agent must satisfy the basic logic rules, this paper incorporates the mechanism knowledge in order to speed up the efficiency of the policy search.

#### Environment agent design

##### Markov decision process

The dynamic interaction process between the environment agent and the ego vehicle is constructed as a Markov decision process (MDP), which can be defined by the following 5 tuples:

$$M=\left(S,A,P,\pi ,R\right),$$ (2)

where S is the state space, A is the action space, P is the probabilistic model of state transition, π is the policy model, and R is the reward.

The core content of the MDP is the Markov property. It states that the future state of a system depends solely on its current state, independent of the sequence of states that led to the current state. In other words, the next state in the sequence is completely determined by the current state and is not affected by the sequence of previous states. The Markov property can be expressed as

pst+1st=pst+1ht,pst+1|st,at=pst+1|ht,at, (3)

where the history of states is $h_{t}=\left\{s_{1},s_{2},s_{3},\ldots ,s_{t}\right\}$.

##### Soft actor critic

RL is a powerful tool for solving MDPs and find optimal or suboptimal policies for learning agents. The SAC algorithm [28] is a widely used RL algorithm. It is a model-free, off-policy algorithm based on an actor-critic framework. SAC uses stochastic policy, which can make the policy as random as possible. The agent can explore the state space *S* more fully, avoid the policy falling into the local optimum early, and can explore multiple feasible solutions to complete the specified task to improve anti-interference ability. It should be noted that the proposed algorithm is not limited to the use of the SAC algorithm, but other novel RL methods can be also applied to the method.

According to MDP, SAC seeks to solve the following maximum entropy problem:

$$\begin{array}{c} \pi^{*}=\text{arg}{\underset{\pi }{\text{max}}}_{\left(s_{t},a_{t}\right)∼\rho_{\pi }}\left[\sum_{t}r\left(s_{t},a_{t}\right)+\alpha H\left(\pi \left(\cdot |s_{t}\right)\right)\right], \end{array}$$ (4)

where H is entropy and α is the temperature parameter. With α→0, the maximum entropy RL gradually approaches the conventional RL.

The basic flow of soft policy iteration is to run 2 steps of policy evaluation and policy improvement alternately until convergence. In the policy evaluation step of soft policy iteration, the value of policy π is calculated according to the maximum entropy objective. The soft *Q* value can be obtained by iterating the Behrman backup operator, that is,

$$T^{\pi }Q\left(s_{t},a_{t}\right)\overset{\Delta }{=}r\left(s_{t},a_{t}\right)+\gamma_{s_{t+1}∼p}\left[V\left(s_{t+1}\right)\right]$$ (5)

The goal of soft policy improvement is to search for a new policy $\pi_{\mathrm{new}}$ that is better than the current policy $\pi_{\mathrm{old}}$. To achieve this goal, we can represent the policy as a Gaussian distribution and reduce the gap between the current policy and the new policy by minimizing the Kullback–Leibler divergence.

$$\pi_{\text{new}}=\text{arg}\underset{\pi^{\prime }\in \Pi }{\text{min}}D_{\text{KL}}\left(\pi^{\prime }\left(\cdot |s_{t}\right)∥\frac{\text{exp}\left(Q^{\pi_{\text{old}}}\left(s_{t},\cdot \right)\right)}{Z^{\pi_{\text{old}}}\left(s_{t}\right)}\right),$$ (6)

where $Z^{\pi_{\text{old}}}\left(s_{t}\right)$ is the normalized distribution of Q values; it will not contribute to the policy gradient, so it can be ignored.

Soft policy iteration has convergence and optimality; see Haarnoja et al. [29].

##### State and action space design

During the normal operation of autonomous vehicles, a sudden cut-in of environment vehicles is a common risky behavior that may lead to emergency situations and jeopardize traffic safety. First, a sudden cut-in may disrupt the autonomous vehicle’s path and speed planning, requiring the system to react quickly to avoid collisions or violations. Second, sudden engagement can trigger emergency braking or evasive maneuvers, and the braking system and evasive strategies need time to respond, potentially resulting in collisions with rear-end vehicles or roadway blockages that can lead to traffic congestion and more serious accidents.

**Fig. 6.**
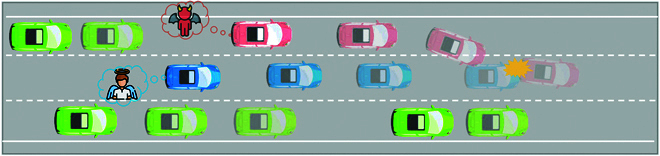
Adversarial behavior of the environment agent. The blue car represents the ego vehicle driving normally, and the red car represents the environment agent with adversarial behavior.

Figure 6 presents a schematic of the environment agent generating an adversarial behavior. Among them, the blue car represents the ego vehicle and the red car represents the environment agent. When the ego vehicle is driving normally, the environment agent will look for a time to cut in from the neighboring lanes to the current lane and minimize the ego vehicle’s performance as much as possible through the control of the throttle, brake, and steering, so as to maximize the risk of side collision, corner collision, or front collision.

The state design is required to fully consider the input information required by the environment agent. Define the state space S as follows:

$$S=\left[\Delta s\;\Delta d\;\begin{array}{ccc} \Delta v_{s} & v_{s} & a_{s} \end{array}\right],$$ (7)

where Δs is the relative longitudinal distance between the environment agent and the ego vehicle, Δd is the relative lateral distance, $\Delta v_{s}$ is the relative longitudinal velocity, and $v_{s}$ and $a_{s}$ are the longitudinal velocity and the longitudinal acceleration of the environment agent, respectively.

The underlying principle of the adversarial policy generated by the environment agent is that the agent should be as close to the ego vehicle as possible to maximize the risk of collision. Therefore, the behavior toward the environment agent moving away from the ego vehicle is against the basic rules of logic. To solve this problem, this paper combines the trajectory planning method based on mechanism rules to construct the RL problem and the action space.

**Fig. 7.**
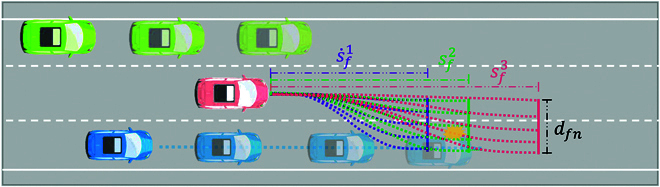
Construction of the adversarial policy search problem for the environment agent based on polynomial curves.

As shown in Fig. 7, the quintic and quartic polynomial curves are respectively applied to describe the longitudinal and lateral trajectory planning process of the environment agent [30]:

$$\left[\begin{array}{cccc} \begin{array}{c} 1\\ 0\\ 0\\ \begin{array}{c} 1\\ 0\\ 0 \end{array} \end{array} & \begin{array}{c} \begin{array}{c} 0\\ 1\\ 0 \end{array}\\ t\\ 1\\ 0 \end{array} & \begin{array}{c} \begin{array}{c} 0\\ 0\\ 2 \end{array}\\ t^{2}\\ 2t\\ 2 \end{array} & \begin{array}{ccc} \begin{array}{c} \begin{array}{c} 0\\ 0\\ 0 \end{array}\\ t^{3}\\ 3t^{2}\\ 6t \end{array} & \begin{array}{c} \begin{array}{c} 0\\ 0\\ 0 \end{array}\\ t^{4}\\ 4t^{3}\\ 12t^{2} \end{array} & \begin{array}{c} \begin{array}{c} 0\\ 0\\ 0 \end{array}\\ t^{5}\\ 5t^{4}\\ 20t^{3} \end{array} \end{array} \end{array}\right]\cdot p_{d}=\left[\begin{array}{c} \begin{array}{c} d_{0}\\ {\overset{\cdot }{d}}_{0}\\ {\ddot{d}}_{0} \end{array}\\ d_{\mathrm{fn}}\\ {\overset{\cdot }{d}}_{\mathrm{fn}}\\ {\ddot{d}}_{\mathrm{fn}} \end{array}\right],$$ (8)

$$\left[\begin{array}{lllll} 1 & 0 & 0 & 0 & 0\\ 0 & 1 & 0 & 0 & 0\\ 0 & 0 & 2 & 0 & 0\\ 0 & 1 & 2t & 3t^{2} & 4t^{3}\\ 0 & 0 & 2 & 6t & 12t^{2} \end{array}\right]\cdot p_{s}=\left[\begin{array}{l} s_{0}\\ {\overset{\cdot }{s}}_{0}\\ {\ddot{s}}_{0}\\ {\overset{\cdot }{s}}_{f}\\ {\ddot{s}}_{f} \end{array}\right],$$ (9)

$$p_{d}={\left[\begin{array}{llllll} a_{d}^{0} & a_{d}^{1} & a_{d}^{2} & a_{d}^{3} & a_{d}^{4} & a_{d}^{5} \end{array}\right]}^{T},$$ (10)

$$p_{s}={\left[\begin{array}{lllll} a_{s}^{0} & a_{s}^{1} & a_{s}^{2} & a_{s}^{3} & a_{s}^{4} \end{array}\right]}^{T},$$ (11)

where t is the time variable, $p_{d}$ represents the coefficients of quintic polynomials for lateral planning, ${\left[\begin{array}{llllll} d_{0} & {\overset{\cdot }{d}}_{0} & {\ddot{d}}_{0} & d_{\mathrm{fn}} & {\overset{\cdot }{d}}_{\mathrm{fn}} & {\ddot{d}}_{\mathrm{fn}} \end{array}\right]}^{T}$ is the boundary condition of a quintic polynomial, $p_{s}$ represents the coefficients of quartic polynomials for longitudinal planning, and ${\left[\begin{array}{lllll} s_{0} & {\overset{\cdot }{s}}_{0} & {\ddot{s}}_{0} & {\overset{\cdot }{s}}_{f} & {\ddot{s}}_{f} \end{array}\right]}^{T}$ is the boundary condition of a quartic polynomial. In the Frenet coordinate system [31], s represents the arc length along the road, used for longitudinal position description, while the lateral position is represented by the offset d perpendicular to the path. The polynomial coefficients $p_{d}$ and $p_{s}$ can be solved by substituting the planning time $T_{c}$ into the polynomial planning equations. Specifically, the parameters $d_{0}$, ${\overset{\cdot }{d}}_{0}$, and ${\ddot{d}}_{0}$ represent the initial position, initial velocity, and initial acceleration, respectively; $d_{\mathrm{fn}}$, ${\overset{\cdot }{d}}_{\mathrm{fn}}$, and ${\ddot{d}}_{\mathrm{fn}}$ represent the final position, final velocity, and final acceleration, respectively. Similarly, the parameters $s_{0}$, ${\overset{\cdot }{s}}_{0}$, and ${\ddot{s}}_{0}$ represent the initial position, initial velocity, and initial acceleration, respectively; $s_{f}$, ${\overset{\cdot }{s}}_{f}$, and ${\ddot{s}}_{f}$ represent the final position, final velocity, and final acceleration, respectively. $a_{d}^{i}$ (*i* = 0, 1, 2, 3, 4, 5) are the coefficients of the quintic polynomial, and $a_{s}^{i}$ (*i* = 0, 1, 2, 3, 4) are the coefficients of the quartic polynomial. Using the initial and final boundary conditions in the above matrix equations, the polynomial coefficients can be solved to obtain the polynomial forms for trajectory planning. The quintic polynomial is used for lateral planning, and the quartic polynomial is used for longitudinal planning.

The design of the action space needs to consider the goal of the autonomous driving task. The action space of the RL problem is designed as follows:

$$A=\left[\begin{array}{cc} d_{\mathrm{fn}} & {\overset{\cdot }{s}}_{f} \end{array}\right],$$ (12)

where $d_{\mathrm{fn}}$ and ${\overset{\cdot }{s}}_{f}$ are the expected lateral offset and expected longitudinal velocity after time $T_{c}$. A simple proportional–integral–derivative controller is applied for tracking the desired trajectory generated by the RL algorithm [32].

##### Reward design

The design of the reward function is very important for RL algorithms. The reward function is used to guide and evaluate the agent’s behavior, which has a direct impact on the performance of the algorithm. In this paper, the reward function with adversarial nature is designed to approach the performance boundary of the ego vehicle by guiding the environment agent to continuously generate adversarial behaviors.

Firstly, the adversarial policy of the environment agent should improve the collision risk with the ego vehicle as much as possible. The artificial potential field method is a kind of virtual force method [33], the basic idea of which is to design the movement of the robot in the surrounding environment into an abstract artificial gravitational field. The artificial potential field contains 2 kinds of force fields: the attractive field formed by the position of the moving target and the repulsive field formed by the obstacle. Therefore, inspired by the improved artificial potential field theory [34], the collision risk of the ego vehicle is quantified.

The risk reward $r_{1}$ is designed as follows:

$$\begin{array}{c} \begin{array}{c} r_{1}=-\sum_{i=1}^{m}\text{min}\left(\text{exp}\left(\frac{\left(\text{min}{\left(\text{max}\left(\Delta d-L_{a},r_{\text{min}}\right),r_{\text{max}}\right)}^{2}\right.}{\delta_{1}^{2}}\right.\right.\\ +\left.\left.\frac{\left(\text{min}{\left(\text{max}\left(\Delta s-L_{b},r_{\text{min}}\right),r_{\text{max}}\right)}^{2}\right.}{\delta_{2}^{2}}\right)\cdot k_{f}\cdot g+b,0\right) \end{array} \end{array}$$ (13)

where $r_{\text{min}}$ and $r_{\text{max}}$ are the safe distance penalty lower bound constant and the safe distance penalty upper bound constant, respectively. $\delta_{1}$ and $\delta_{2}$ are the lateral repulsion influence factor and the longitudinal repulsion influence factor, respectively. $L_{a}$ is the vehicle width, $L_{b}$ is the vehicle length, and $k_{f}$ is the scale factor. In order to keep the reward value in a reasonable range, g is defined as the linear mapping proportion and b is defined as the linear mapping deviation. g and b can be expressed as follows:

$$g=-\frac{\rho_{n}}{k_{f}\cdot S-k_{f}},$$ (14)

$$b=\rho_{n}-g\cdot k_{f},$$ (15)

where $\rho_{n}$ is the safe distance penalty factor. S is the proportional correction factor, which can be expressed as

$$S=\text{exp}\left(\frac{{\left(d_{\text{thre}}-L_{a}\right)}^{2}}{\delta_{1}^{2}}+\frac{{\left(s_{\text{thre}}-L_{b}\right)}^{2}}{\delta_{2}^{2}}\right),$$ (16)

where $d_{\text{thre}}$ and $s_{\text{thre}}$ are the lateral and longitudinal threshold values of the safe distance penalty, respectively.

The vehicle speed is encouraged to be maintained within a reasonable range. In the start-up stage, the reward function is designed to guide the vehicle to accelerate from 0, while in the speed maintenance stage, the reward function is designed to maintain in a preset speed interval. In order to design the adversarial reward to harm the longitudinal performance of the vehicle, the minimum speed reward $r_{2}$ is designed as follows:

$$r_{2}=-\rho_{L}\times \kappa_{L}-\rho_{H}\times \kappa_{H},$$ (17)

where $\rho_{L}$ is the start-up stage reward factor, $\rho_{H}$ is the speed maintenance stage reward factor, $\kappa_{L}=v_{s}/v_{\text{min}}$ is the ratio between the longitudinal velocity $v_{s}$ and the desired minimum velocity $v_{\min}$, and $\kappa_{H}=\left(v_{s}-v_{\text{min}}\right)/\left(v_{\text{max}}-v_{\text{min}}\right)$ is the ratio of the vehicle speed $v_{s}$ to the desired maximum speed $v_{\max}$ based on the desired minimum speed $v_{\min}$.

Causing the collision accident is considered to be the most serious consequence that can result from the adversarial behavior produced by the environment agent. Therefore, when the collision occurs, the collision reward $r_{3}$ is set as follows.

$$r_{3}=\left\{\begin{array}{cc} 0 & \text{not collision},\\ \rho_{\text{coll}} & \;\text{collision}, \end{array}\right.$$ (18)

where $\rho_{\text{coll}}$ is the collision reward factor. $\rho_{\text{coll}}$ is set to a positive value to indicate that policies that receive this reward are encouraged.

The total reward function is

$$R=-r_{1}+r_{2}+r_{3}$$ (19)

All reward function parameters are listed in Table 2.

**Table 2.** Reward function parameters setting

Reward function parameter terms	Symbol and value
Safe distance penalty lower bound constant	$r_{\text{min}}=0.0$
Safe distance penalty upper bound constant	$r_{\text{max}}=150.0$
Lateral repulsion influence factor	$\delta_{1}=8.0$
Longitudinal repulsion influence factor	$\delta_{2}=10.0$
Scale factor	$k_{f}=0.001$
Vehicle width	$L_{a}=2.077\;m$
Vehicle length	$L_{b}=5.037\;m$
Safe distance penalty factor	$\rho_{n}=-18.0$
Lateral threshold value of the safe distance penalty	$d_{\text{thre}}=0.8$
Longitudinal threshold value of the safe distance penalty	$S_{\text{thre}}=20.0$
Start-up stage reward factor	$\rho_{L}=0.5$
Speed maintenance stage reward factor	$\rho_{H}=4.0$
Expected minimum speed	$v_{\text{min}}=7.5\;m/s$
Expected maximum speed	$v_{\text{max}}=22.0\;m/s$
Collision reward factor	$\rho_{\text{coll}}=200.0$

### Data generation of varied difficulty scenarios

In the “Environment agent policy search” section, we introduce the concept of environment agent to realize the adversarial policy search by combining logic rules with RL. However, due to the black-box nature of data-driven methods, while adversarial actions can be generated, the difficulty of generating adversarial actions is difficult to quantify accurately, which limits the rationality of adversarial scenario generation.

In this section, a data generation method based on scenarios of varying difficulty is presented. The method uses the performance of different stages in the policy search convergence process as a reference to quantify the adversarial intensity, thereby achieving a quantitative representation of scenario difficulty. The model parameters of the environment agent trained on different stages are updated and saved and then output to the constructed policy group. The policy group is used to generate data that forms the basis for training the scenario difficulty quantitative representation model.

#### Policy group construction

**Fig. 8.**
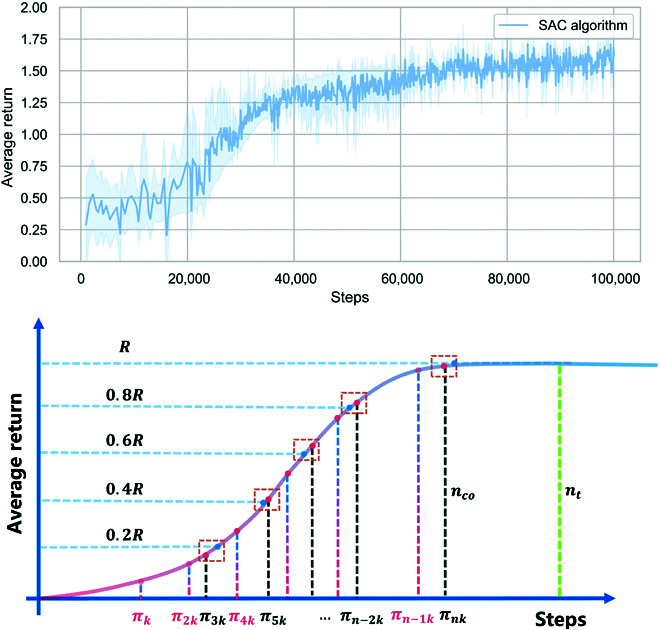
Construction of the policy group. SAC, soft actor critic.

As shown in Fig. 8, the schematic of the average return during the policy search of the environment agent is presented. The horizontal axis shows the number of training steps, and the vertical axis shows the average return. The training details can be seen in Results and Discussion.

An RL training process with stable convergence can be divided into 2 phases, i.e., the performance improvement phase and the convergence stabilization phase. In the performance improvement phase, the average return is still continuously increasing, which indicates that the policy search is still ongoing and the model parameters are still being updated to produce a better performance. In the convergence stabilization phase, however, the average return remains basically unchanged, indicating that the policy search is basically over, and the obtained policy is already the optimal policy that the current algorithm can achieve.

Considering that the intensity of the environment agent’s adversarial behavior against the ego vehicle may increase nonlinearly with the training process, a policy filtering method is proposed to ensure that the policies within the policy group can generate distinguishable and reasonable adversarial scenarios with different intensities. The policy filtering algorithm is shown in Algorithm 1 and Fig. 8.

The initialization and parameter setting of the algorithm are performed in lines 1 to 3. In lines 4 and 5, through trial training of the environment agent, reasonable total steps $n_{t}$, performance improvement steps $n_{\mathrm{co}}$, and maximum average return R are obtained.

The policy search of the environment agent $\pi_{E}$ is shown in line 7, and the parameter θ of $\pi_{E}$ is updated using Eq. 4. Lines 8 to 12 represent the sampling operations conducted on model parameters during the convergence process of the algorithm. The sampled model parameters are placed in set Ξ. The sampling interval is k; i.e., the model is sampled every k rounds. The sampled model $\pi_{k}$ and the corresponding training steps $e_{k}$ form a binary group and are placed in set Ξ, which can be denoted as

$$\Xi =\left\{\Pi_{k},\Pi_{2k},\Pi_{3k},\ldots ,\Pi_{\mathrm{nk}}\right\},$$ (20)

where $\Pi_{k}=\left\{\pi_{k},e_{k}\right\}$, $n=\mathrm{len}\left(\Xi \right)$.

As shown in Fig. 8, the bottom of the horizontal axis represents the sampling models $\pi_{k},\pi_{2k},\ldots ,\pi_{\mathrm{nk}}$ at different phases in set Ξ, corresponding to the positions at different time steps $e_{k},e_{2k},\ldots ,e_{\mathrm{nk}}$.


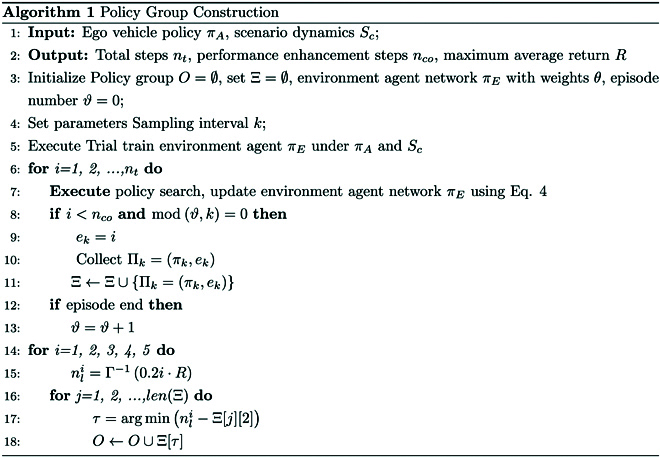


In lines 13 to 17, policy filtering is performed to ensure that the intensity of the adversarial behaviors produced by the environment agent models in policy group O grows linearly. To quantify the intensity of adversarial strength, the maximum average return R is used. Figure 8 illustrates the policy filtering process. First, the mapping relation between the average return curve and the training steps is defined as

$$R_{s}=\Gamma \left(n_{s}\right),$$ (21)

where $R_{s}$ is the corresponding average return value at $n_{s}$ steps.

In lines 13 and 14, the maximum average return R is linearly split, as shown in Fig. 8, to obtain the number of training steps corresponding to the dividing line (the horizontal coordinates corresponding to the blue circle center point). In lines 15 and 16, a find algorithm is executed to find the index τ of the sampled model that is closest to $n_{l}^{i}$ in $e_{k},e_{2k},\ldots ,e_{\mathrm{nk}}$ (i.e., closest pink center point to the blue center point in Fig. 8).

In line 17, the sampling models are extracted from set Ξ according to index τ and deposited into O to finalize the construction of the policy group. The sampled models that have been filtered and deposited into policy group O are shown as black dashed lines in Fig. 8.

#### Varied difficulty scenario dataset

The constructed policy group O is used to build a varied difficulty scenario dataset, and this process is carried out in a simulation environment. The parameters of the simulation environment are randomly configured, which is to simulate more diverse real-world inputs and ensure the generalization of subsequent model training.

The pseudocode of the construction for the varied difficulty scenario dataset is shown in Algorithm 2.


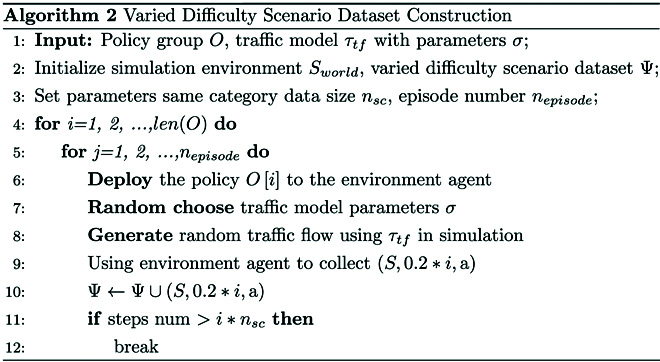


First, line 1 initializes the simulation environment $S_{\text{world}}$ and varied difficulty scenario dataset Ψ. Lines 2 and 3 determine the input and parameter setting of the algorithm. Lines 4 to 12 represent a complete dataset construction process. In line 6, the policies in the policy group O are deployed to the environment agent. In lines 7 and 8, the random traffic flow $\tau_{\mathrm{tf}}$ is generated in the simulation environment by randomizing the parameters of the traffic model σ. In lines 9 and 10, the environment agent is used to collect the ternary $\left(S,0.2*i,a\right)$ into Ψ, where 0.2∗i denotes the difficulty factor of the scenario. Lines 11 and 12 indicate that when the collected data size of the same category exceeds $n_{\mathrm{sc}}$, the data collection for the corresponding difficulty level is finished. The algorithm then jumps out the current inner loop, proceeds to deploy the environment agent’s policy for the next difficulty level, and continues until varied difficulty scenario datasets Ψ are completely collected.

### Quantitative representation of scenario difficulty

To obtain an environment agent model with an adversarial behavior, a quantitative representation model is constructed. The model is based on a deep neural network with a transformer decoding architecture. As shown in Fig. 5, the input of the model is the state s and the scenario difficulty factor $X_{\mathrm{scd}}$, and the output is the action a of the environment agent.

In recent years, the transformer model has gained much attention [35,36], and its core idea is the self-attention mechanism. The key to the self-attention mechanism is to assign different weights to certain positions, thus enabling the model to better capture long-distance dependencies and global information in the input sequence. This mechanism not only contributes to a deeper understanding of the intrinsic structure of the sequence but also provides interpretability for the model, allowing it to focus on important parts of the sequence.

The proposed quantitative mode is constructed based on the transformer encoder model [37]. The transformer encoder consists of alternating layers of self-attention and multilayer perceptron block. Layer norm (LN) is applied before every block, and residual connections after every block.

The input of the neural network includes the scenario difficulty factor $X_{\mathrm{scd}}$ and the relative information between the environment agent and the ego vehicle. The input vector can be expressed as follows:

$$X=\left[X_{\mathrm{scd}},s\right]=\left[X_{\mathrm{scd}},\Delta s,\Delta d,\Delta v_{s},v_{s}a_{s}\right]$$ (22)

The standard transformer model takes a 1-dimensional sequence of token embeddings as input. The embedding projection is then applied to the extracted features from the input vector.

$$h_{0}=\left[X_{\mathrm{scd}}E;\Delta sE;\Delta dE,\Delta v_{s}E,v_{s}E,a_{s}E\right],\;E\in^{\;P\times D}$$ (23)

The transformer encoder model can be expressed as

$$z_{\ell }^{\prime }=\text{MSA}\left(\text{LN}\left(z_{\ell -1}\right)\right)+z_{\ell -1}\;\ell =1\ldots L$$ (24)

$$y=\text{LN}\left(z_{L}^{0}\right)$$ (25)

In Eq. 24, the self-attention criterion divides the input embedding into 3 vectors V, K, and Q [38]. The scaled dot-product attention is calculated according to Eq. 26.

$$\Theta =\text{Attention}\left(Q,K,V\right)=\text{softmax}\left(\frac{{\mathrm{QK}}^{T}}{\sqrt{d_{k}}}\right)V,$$ (26)

where Θ is the score matrix, Q is a query vector, K is a key vector, V is a value vector, and $d_{k}$ is a normalization.

The training process of scenario difficulty quantitative representation model φ is shown in Algorithm 3. φ is trained with data from varied difficulty scenario dataset Ψ. In line 5, a batch B is randomly sampled from φ. In lines 7 and 8, the loss function is calculated by the mean square error between the predicted and true values, where a and $\hat{a}$ denote the ground truth and predicted values, respectively. The model is trained using the Adams optimizer.

The score matrix of the transformer can be used to analyze the focus of the model when processing sequences, thus improving the interpretability of the model’s behavior. The score matrix Θ can calculated using Eq. 26.


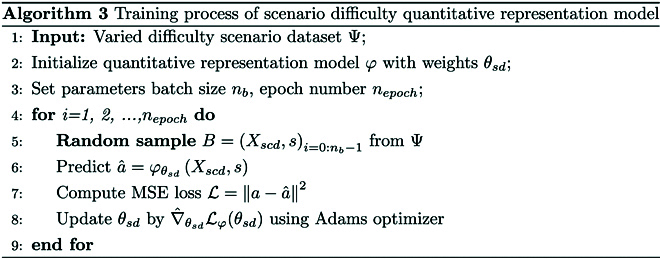

$$\Theta =\left[\begin{array}{llll} \mu_{\mathrm{scd}}^{1\times 1} & \mu_{S_{\left[1\right]}}^{1\times 2} & \cdots & \mu_{S_{\left[n\right]}}^{1\times \left(n+1\right)}\\ \mu_{S_{\left[1\right]}}^{2\times 1} & \mu_{a}^{2\times 2} & \cdots & \mu_{a}^{2\times \left(n+1\right)}\\ \vdots & \vdots & \ddots & \vdots \\ \mu_{S_{\left[n\right]}}^{n+1\times 1} & \mu_{a}^{n+1\times 2} & \cdots & \mu_{a}^{n+1\times \left(n+1\right)} \end{array}\right],$$ (27)

where μ is a scalar and the bottom-right symbol is used to distinguish the source of μ. Θ can be used to characterize the feature correlation between each state. For the scenario difficulty quantitative representation model, the state correlation between the scenario difficulty factor $X_{\mathrm{scd}}$ and the relative information between the environment agent and the ego vehicle is the most noteworthy. By summing the elements along columns in matrix Θ, a new matrix ${\tilde{\Theta }}^{1\times \left(n+1\right)}$ can be obtained.

In order to obtain an ordering of the characteristic correlation between the scenario difficulty factor $X_{\mathrm{scd}}$ and the other states s, ${\tilde{\Theta }}^{1\times \left(n+1\right)}$ is sorted and the indexes of the array elements sorted are obtained, as shown in Eq. 28:

$$\Lambda =\text{argsort}\left({\tilde{\Theta }}^{\left(n+1\right)\times \left(n+1\right)}\right),$$ (28)

where the index matrix Λ is the vector of 1×n and the corresponding sorted feature contribution value is $ϒ={\tilde{\Theta }}^{1\times \left(n+1\right)}\left[\Lambda \right]$.

## Data Availability

The data that support the findings of this study are available from the School of Automotive Studies, Tongji University. Restrictions apply to the availability of these data, which were used under license for this study. Data access inquiries can be sent to shuo_yang@tongji.edu.cn.
